# Impaired dendritic cell maturation and IL-10 production following *H. pylori* stimulation in gastric cancer patients

**DOI:** 10.1007/s00253-012-4034-z

**Published:** 2012-04-13

**Authors:** Lin-Li Chang, Sheng-Wen Wang, I-Chen Wu, Fang-Jung Yu, Yu-Chung Su, Ye-Pin Chen, Deng-Chyang Wu, Chang-Hung Kuo, Chih-Hsing Hung

**Affiliations:** 1 Department of Microbiology, Kaohsiung Medical University, 100 Shih-Chuan 1st Road, Kaohsiung, 807 Taiwan; 2 Graduate Institute of Medicine, Kaohsiung Medical University, Kaohsiung, Taiwan; 3 Division of Gastroenterology, Department of Internal Medicine, Kaohsiung Medical University Hospital, 100 Shih-Chuan 1 st Road, Kaohsiung, 807 Taiwan; 4 Department of Pediatrics, Faculty of Pediatrics, College of Medicine, Kaohsiung Medical University Hospital, 100 Shih-Chuan 1 st Road, Kaohsiung, 807 Taiwan; 5 Department of Pediatrics, Kaohsiung Medical University Hospital, Kaohsiung, Taiwan; 6 Department of Pediatrics, Kaohsiung Municipal Ta-Tung Hospital, Kaohsiung, Taiwan

**Keywords:** Dendritic cell, Epigenetics, Gastric cancer, *Helicobacter pylori*, IL-10

## Abstract

The current study was to investigate the interaction between *Helicobacter pylori* and human dendritic cells (DCs). Whether impaired DC function can influence the outcome of *H. pylori* infections. Human monocyte-derived DCs (MDDCs) from five gastric cancer patients and nine healthy controls were stimulated with *H. pylori*. Maturation markers of MDDC were examined by flow cytometry. IL-10 and TNF-α released by MDDCs and IL-17 produced by T cells were measured by ELISA. Regulatory signaling pathways of IL-10 were examined by ELISA, western blotting, and chromatin immunoprecipitation assay. The results showed that as compared with healthy individuals, the maturation marker CD40 in MDDCs, IL-17A expression from T cells, and IL-10 expression from MDDCs were significantly lower in gastric cancer patients. Blocking DC-SIGN, TLR2, and TLR4 could reverse *H. pylori*-associated IL-10 production. Activation of the p38 MAPK and NF-kB signaling pathways concomitant with decreased tri-methylated H3K9 and increased acetylated H3 accounted for the effect of *H. pylori* on IL-10 expression. Furthermore, upregulated IL-10 expression was significantly suppressed in *H. pylori*-pulsed MDDCs by histone acetyltransferase and methyltransferase inhibitors. Taken together, impaired DC function contributes to the less effective innate and adaptive immune responses against *H. pylori* seen in gastric cancer patients*. H. pylori* can regulate IL-10 production through Toll-like and DC-SIGN receptors, activates p-p38 MAPK signaling and the transcription factors NF-kB, and modulates histone modification.

## Introduction

*Helicobacter pylori* is a Gram-negative, spiral-shaped bacterium that can colonize the human gastric mucosa. *H. pylori* infections are highly prevalent and a wide range of clinical manifestations have been observed. Most people infected with *H. pylori* have asymptomatic gastritis, but approximately 10% of infected patients develop gastric or duodenal ulcers, and 1% develop gastric adenocarcinoma (Fock and Ang 2010). These diverse outcomes have been attributed to the interplay of several factors, including *H. pylori* virulence, host genetic susceptibility and local immune responses, and environmental conditions. The VacA (vacuolating cytotoxin A) and the Cag (cytotoxin-associated gene) pathogenicity island of *H. pylori* have been shown to cause adverse outcomes, but these factors are not predictive of the development of gastric cancer in *H. pylori*-infected patients (Backert and Selbach 2008).

The host immune response to the bacterium is an intrinsic component in the pathogenesis of these diseases, and growing evidence indicates that dendritic cells (DCs) are involved in the response to *H. pylori* infection (Andres et al. 2011; Bimczok et al. 2010; Necchi et al. 2009). DCs are professional antigen-presenting cells with critical roles in the initiation and progression of innate and adaptive immunity (Banchereau et al. 2000). Mouse studies have shown that DCs are recruited to the gastric mucosa after *H. pylori* infection (Kao et al. 2006, 2010). Using two photon and confocal microscopy, Rescigno et al. localized DCs to close to the surface epithelium of the normal intestine and also showed that their cellular processes extend into the epithelial layer (Rescigno et al. 2001).

DCs are also known to traverse the gut epithelium tight junction to sample luminal bacteria (Ito et al. 2008) and deliver them to nearby lymph nodes, where they then activate naïve T cells and direct the T-cell response, according to their state of activation (Ismail et al. 2003; Lundgren et al. 2005; Sansonetti and Di Santo 2007). These data suggest that DCs are a front line of host immune responses to *H. pylori* infection and play a central role in determining whether an infected host clears the bacteria or goes on to develop chronic inflammation.

Although the precise mechanisms by which *H. pylori* induces carcinogenesis are still unclear, inflammation is the most commonly cited factor in the carcinogenic process. Various cytokines produced by DCs are known to affect the outcome of subsequent T-cell activation: TNF-α, IFN-γ, and IL-12 drive Th1 responses while IL-4, IL-10, and IL-13 promote Th2/Treg responses (Moser and Murphy 2000). The accumulation of *H. pylori*-specific CD4^+^ T cells observed in infected human gastric mucosa suggests that CD4^+^ T-cell-mediated Th1 immune responses may be critical in the development of *H. pylori*-induced gastritis (Bimczok et al. 2010). The cytokine IL-10, which has potent anti-inflammatory and immune regulatory properties, inhibits various inflammatory cytokines, including IL-12, and may down-regulate host Th1-type responses to gastric *H. pylori* infection. IL-10 knockout mice infected with *Helicobacter felis* demonstrate that a lack of IL-10 causes severe chronic gastritis and substantially enhances the *Helicobacter*-specific Th1 immune response (Ismail et al. 2003). Although the role of TLRs (Toll-like receptors) in the macrophage response to *H. pylori* infection has been studied intensively, only a few reports concerning the role of TLRs and DC-SIGN (Dendritic Cell-Specific Intercellular adhesion molecule 3-Grabbing Non-integrin) receptors in DCs during *H. pylori* infections (Bergman et al. 2004; Gringhuis et al. 2009).

This study investigated the effects of *H. pylori* on DC activation and maturation and the functional consequences of these effects on naïve T cells. We also examined the regulatory signal pathways in IL-10 cytokine production after *H. pylori* stimulation and compared the cytokine profiles of healthy individuals and gastric cancer patients after stimulation with *H. pylori*. Histone remodeling by bacterial and viral pathogens has recently been shown to influence immune responses during infection (Li et al. 2007), and we therefore also analyzed epigenetic effects during *H. pylori* infections.

## Materials and methods

### Bacteria and growth conditions

*H. pylori* strain ATCC 26695 was inoculated on CDC culture medium (BBL Microbiology System, Cockeysville, MD, USA) for 3 days under micro-aerophilic conditions (12% CO_2_ and 5% O_2_) at 37 °C. *H. pylori* was identified by colony morphology and through positive biochemical tests for ureases, catalase, and oxidase. The characteristic curvy shape of *H. pylori* was also observed under microscope by Gram staining. For further experiments, *H. pylori* was prepared in 0.9% saline solution at a concentration of 1 × 10^9^ bacteria/ml, which was measured by optical density determination at 550 nm and adjusted to a final absorbance of 0.75.

### Monocyte-derived dendritic cell (MDDC) isolation, culture, and treatment with *H. pylori*

The study of human subjects was approved by the Institutional Review Board of Kaohsiung Medical University, Taiwan. After obtaining informed consent, 150–200 ml peripheral blood were obtained and peripheral blood mononuclear cells (PBMCs) were isolated with Ficoll-Paque (Pharmacia Biotech, Uppsala, Sweden) from healthy individuals with no history of *H. pylori* infection (*n* = 9) and from gastric cancer patients (*n* = 5). Blood monocytes were magnetically sorted with CD14 isolation kits (Miltenyi Biotec, Gmbh Bergisch Gladbach, Germany), following the manufacturer’s instructions. MDDCs were obtained by culturing purified monocytes in RPMI 1640 medium containing 10% FCS, 100 IU/ml penicillin, 0.1 mg/ml streptomycin, 50 ng/ml granulocyte–macrophage colony-stimulating factor (GM-CSF; BD Pharmingen, San Diego, CA, USA), and 10 ng/ml IL-4 at 37 °C for 7 days. Purified MDDCs (2 × 10^5^ cells) from healthy individuals and patients were treated with various doses of *H. pylori* (multiplicity of infection, MOI = 1:100 or 1:200) and incubated for 6, 24, or 48 h, depending on the experiment. To examine the involvement of the DC-SIGN and TLRs, MDDCs were pre-treated with anti-DC-SIGN, TLR2, or TLR4 antibodies (BD Biosciences, San Jose, CA, USA) 1 h prior to treatment with *H. pylori*. To determine the roles of the three MAPK (mitogen-activated protein kinases) signaling transduction pathways in IL-10 expression, MDDCs were pre-treated with inhibitors of ERK (extracellular signal-regulated kinases, PD98059), p38MAPK (SB203580), JNK (Jun N-terminal kinase, SP600125), and IκB-alpha phosphorylation (BAY-117082) 1 h before exposure to *H. pylori.* All MAPK and IκB-alpha inhibitors were purchased from Sigma-Aldrich (St. Louis, MO, USA). To evaluate histone modification involving IL-10 expression, MDDCs were treated with histone acetyltransferase inhibitor anacardic acid (AA, 0.2 μg/ml) and histone methyltransferase inhibitor 5′-deoxy-5′-(methylthio) adenosine (MTA, 0.1 μg/ml) (BD Pharmingen). The production of cytokines in the culture supernatants was determined by enzyme-linked immunosorbent assay (ELISA).

### Western blotting

For Western blotting analysis, MDDCs were treated with *H. pylori* (multiplicity of infection, MOI = 1:100 or 1:200) for 1 h and then lysed. Equal quantities of whole cell lysates were analyzed by Western blotting with anti-p65, anti-phospho-p65 (pp65), anti-p38, anti-phospho-p38, anti-ERK, anti-phospho-ERK, anti-JNK, and anti-phospho-JNK antibodies (Santa Cruz Biotechnology, Santa Cruz, CA, USA). Immunoreactive bands were visualized using horseradish peroxidase-conjugated secondary antibodies and the enhanced chemiluminescence (ECL) system (Amersham Pharmacia Biotech, Uppsala, Sweden).

### Naïve T-cell purification and co-culture with *H. pylori*-treated MDDCs

Autologous naïve CD4^+^ T cells were purified from PBMCs by using CD4 magnetic beads (Miltenyi Biotec GmbH, Germany) and activated with CD3 and CD28 antibodies (eBioscience, San Diego, CA, USA). After treatment with *H. pylori* for 48 h, MDDCs were co-cultured with autologous naïve T cells (DC/T cell ratio 1:10, the optimal ratio for cytokine assay) for 7 days. Cell culture supernatants were collected for IL-17A measurement by ELISA assay.

### ELISA for determination of cytokine levels

The presence of IL-10, TNF-α, and IL-17A in culture supernatants was determined by ELISA (R&D Systems, Minneapolis, MN, USA).

### MDDC activation and maturation

MDDCs (5 × 10^5^ cells) were incubated in the presence of 10^8^ CFU *H. pylori* for 48 h in RPMI 1640 medium supplemented with 10% FCS. MDDCs were then stained with the following mAbs to evaluate the maturation by flow cytometry: FITC conjugated anti-HLA-DR, FITC conjugated anti-CD80, FITC conjugated anti-CD40, or FITC conjugated anti-CD86 (all mAbs from BD Biosciences, MA, USA). Surface markers of DCs were analyzed using a FACScan flow cytometer and CellQuest software (Becton Dickinson).

### Chromatin immunoprecipitation assay (ChIP)

Changes in histone modification were quantitatively analyzed by ChIP along the promoter region of the IL-10 genes after stimulation with *H. pylori*. The ChIP assays were performed according to the manufacturer’s protocol with minor modifications. MDDCs (5 × 10^5^ cells) were treated with 1% formaldehyde for 10 min at RT followed by DNA sonication to 200 to 400 bp. Chromatin was immunoprecipitated overnight with antibodies for acetylated histone H3 and H4 and for tri-methylated H3K4, H3K9, H3k36, and H3k79 (Upstate Biotechnology, Waltham, MA, USA). Immune complexes were collected using a protein A slurry (Invitrogen, Carlsbad, CA, USA), and the DNA was reverse cross-linked, extracted, and quantified with SYBR Green-based detection (Applied Biosystems, Foster City, CA, USA) on a TaqMan SDS 7900HT.

The primers used to amplify specific regions of the IL-10 promoter were as follows: IL-10-1, 5′-TATTCTAAGAGAGGTAGCCCATCCT-3′ and 5′-CTTCATTCATTAAAAAGCCACAATC-3′; IL-10-2, 5′-CCGCCTGTACTGTAGGAAGC-3′ and 5′-CCCCAACCTGGGATGAATAC-3′; and GAPDH, 5′-CGGTGCGTGCCCAGTT-3′ and 5′-CCCTACTTTCTCCCCGCTTT-3′. The PCRs were run on an ABI TaqMan SDS 7900HT thermocycler. All TaqMan reagents were purchased from Applied Biosystems. The relative intensities of the amplified products were normalized to the input DNAs and plotted graphically as fold changes.

### Statistical analysis

All data were analyzed by Mann–Whitney tests except for the ChIP assay in which ANOVA tests were used. Data are presented as means ± SE. A *p* value ≤0.05 was considered as a significant difference.

## Results

### A reduced expression of CD40 in *H. pylori*-primed MDDCs from gastric cancer patients

**Fig. 1:**
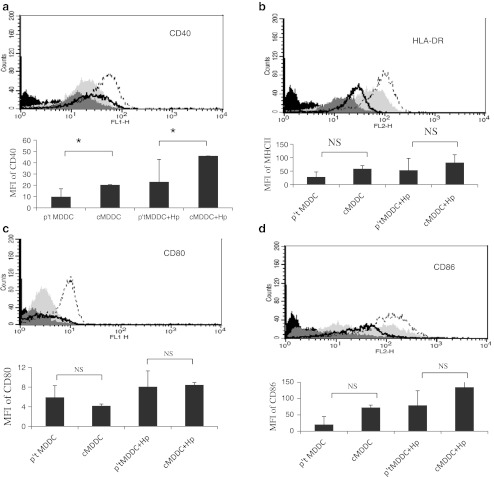
Expression of surface markers on *H. pylori-*primed MDDCs from gastric cancer patients and healthy individuals. Mean fluorescence intensity (MFI) expression of co-stimulatory molecules **a** CD40, **b** HLA-DR, **c** CD80, and **d** CD86 in MDDCs was detected by flow cytometry. CD40 expression was significantly lower in MDDCs from gastric cancer patients. One of three representative experiments is shown. Isotype control: *black*, normal control: *light gray*, patient control: *heavy gray*, normal *H. pylori* 1:200: *black plot line*, patient *H. pylori* 1:200: *black heavy line*. *p = 0.05; *NS* non significant

To examine the effects of *H. pylori* on DC maturation, expression of co-stimulator and antigen-presenting molecules (CD80, CD40, CD86, and HLA-DR) in MDDCs was evaluated by flow cytometry after *H. pylori* stimulation for 48 h. Figure 1 shows that immature MDDCs from gastric cancer patients and healthy individuals significantly increased the surface expression of CD40, CD80, CD86, and HLA-DR following stimulation with *H. pylori*. However, the expression of CD40 co-stimulatory molecules was significantly lower in DCs from gastric cancer patients than in those from healthy individuals, both with and without *H. pylori* stimulation (Fig. 1a).

### Weakened Th17 responses are induced by *H. pylori*-treated MDDCs from gastric cancer patients

Since binding of CD154 (CD40L) on T helper cells to CD40 on MDDC may further activate MDDC and induce a variety of downstream effects, our data suggested that a reduced capacity of MDDC primed by *H. pylori* to express CD40 may weaken the function of MDDC and the communication of MDDC to T helper cells in gastric cancer patients.

**Fig. 2:**
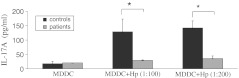
IL-17A expression induced by *H. pylori-*treated MDDCs differs between gastric cancer patients and healthy individuals. Induction of IL-17A production after co-culture of naïve T cell with *H. pylori* pulsed MDDCs from healthy individuals or gastric cancer patients. IL-17A expression differs between these two groups, as measured by ELISA. Data are presented as means and standard deviation of three independent experiments. Mann–Whitney tests were used to calculate statistical significance. *p <0.05; **p <0.01

To characterize the Th response induced by MDDCs pulsed with *H. pylori*, CD4^+^ T cells purified from PBMCs were co-cultured with *H. pylori*-treated MDDCs for 7 days. Supernatant was collected for IL-17A measurement by ELISA. The IL-17A level in MDDCs of healthy individuals and gastric cancer patients challenged with *H. pylori* was significantly higher than in MDDCs which were not pulsed with *H. pylori*. However, IL-17A production was significantly lower in MDDCs from gastric cancer patients than from healthy individuals (Fig. 2). We therefore conclude that immature MDDCs pulsed with *H. pylori* do not induce a robust Th17 response in gastric cancer patients. Since IL-17 is primarily associated with gastric inflammation and targets extracellular pathogens, weakened Th17 response induced by MDDC from gastric cancer patients may contribute to an impaired immunity against extracellular pathogens, thereby favoring bacterial persistence in cancer patients.

### Impaired production of IL-10, but not TNF-α, from MDDCs from gastric cancer patients

**Fig. 3:**
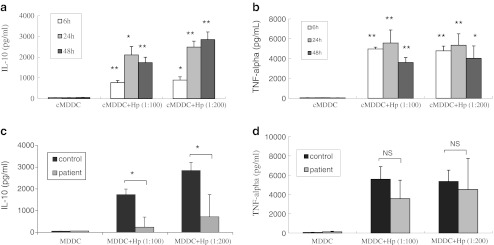
Production of IL-10 and TNF-α from MDDCs after *H. pylori* stimulation. *H. pylori* induces IL-10 (**a**) and TNF-α (**b**) production by MDDCs from healthy individuals in a dose- and time-dependent manner (*n* = 9 individuals). **c** MDDCs from five gastric cancer patients did not produce significant levels of IL-10 after *H. pylori* stimulation, and IL-10 production was significantly lower than in MDDCs from healthy individuals. **d** TNF-α expression did not differ between gastric cancer patients and healthy individuals after *H. pylori* exposure. **p <0.01, *p <0.05 was considered significant versus untreated cells, *NS* non-significant difference. *cMDDC* MDDC from healthy individuals

Stimulation with *H. pylori* induced IL-10 and TNF-α production by MDDCs of healthy individuals in a dose-dependent manner (Fig. 3a, b). Kinetic analysis of cytokine production by MDDCs stimulated with *H. pylori* showed that IL-10 and TNF-α production significantly increased after 6 h and reached saturation levels after 48 and 24 h, respectively (Fig. 3a, b). Analysis of IL-10 production indicated that stimulation with a MOI of 200 (multiplicity of infection of MDDCs—*H. pylori* = 1:200) resulted in maximum cytokine release. Therefore, at 48 h, 200-MOI dose was considered optimal for stimulating IL-10 in subsequent experiments. To compare whether the responses of MDDCs in the presence of *H. pylori* differ between gastric cancer patients and healthy individuals, IL-10 and TNF-α production of MDDCs from gastric cancer patients were also analyzed. Interestingly, MDDCs from gastric cancer patients revealed no significant IL-10 production after *H. pylori* stimulation. Furthermore, IL-10 expression was significantly lower in MDDCs from gastric cancer patients than in MDDCs from healthy individuals (Fig. 3c). However, TNF-α expression did not significantly differ between gastric cancer patients and healthy individuals after *H. pylori* exposure (Fig. 3d).

### *H. pylori* induces IL-10 production via DC-SIGN, TLR2, and TLR4 receptors in MDDCs

**Fig. 4:**
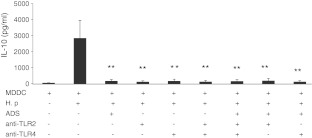
*H. pylori* induced IL-10 production via DC-SIGN, TLR2, and TLR4 receptors in MDDCs. IL-10 expression in *H. pylori-*infected MDDCs from nine healthy individuals in the absence or presence of anti-DC-SIGN, TLR2, or TLR4 antibody, either alone or in the indicated combination for 1 h prior to the treatment of MDDCs with *H. pylori*, was evaluated by ELISA. Statistical significance was determined with one-way ANOVA test. **p <0.01 was considered significant compared with cells not treated with antibody. *TLR2* toll-like receptor 2, *TLR4* toll-like receptor 4

The mechanisms by which IL-10 production is regulated following *H. pylori* infection are not clearly understood. We found that *H. pylori*-induced IL-10 production in MDDCs was significantly suppressed by the addition of anti-DC-SIGN, TLR2, or TLR4 antibodies either alone or in combination before *H. pylori* stimulation (Fig. 4). Notably, these receptor antagonists resulted in similar suppressive effects on IL-10 expression in MDDCs (Fig. 4).

### Production of IL-10 in MDDCs is induced by *H. pylori* via p38 MAPK signaling and NF-kB activation

**Fig. 5:**
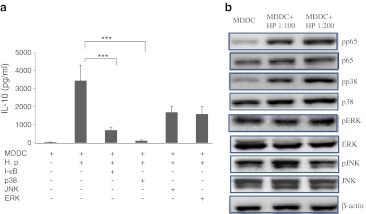
*H. pylori* increases IL-10 production in MDDCs via the p38 MAPK and p65 NF-kB pathways. **a** The level of IL-10 expression in *H. pylori-*infected MDDCs in the presence of 2 μM I-kB inhibitor BAY-117082, p38 phosphorylation inhibitor SB203580, JNK phosphorylation inhibitor SP600125, and ERK phosphorylation inhibitor PD98059. **b** Western blot analyses of phosphorylated p65, p38, ERK, JNK (pp65, pp38, pERK, pJNK), and total p65, p38, ERK, JNK, β-actin was used for internal controls. ***p <0.001

MAPK and NF-kB are important intermediates in TLR-mediated signaling pathways. A p38-MAPK inhibitor (SB203580) and an NF-kB p65 inhibitor (BAY-117082) suppressed *H. pylori*-induced IL-10 expression whereas neither an ERK inhibitor (PD98059) nor a JNK inhibitor (SP600125) had inhibitory effects (Fig. 5a). These data suggest that p38/MAPK and NF-kB activation are critical for sustaining *H. pylori*-mediated IL-10 production. Western blot analysis further confirmed that the increased phosphorylation of p38 and p65 in response to *H. pylori* stimulation activated the MAPK and NF-kB signaling pathways in *H. pylori*-treated MDDCs (Fig. 5b). Notably, *H. pylori* induced IL-10 production through activation of the p38 MAPK and p65 NF-kB pathways but not through activation of the ERK and JNK pathways.

### *H. pylori* regulates IL-10 production through histone modifications

**Fig. 6:**
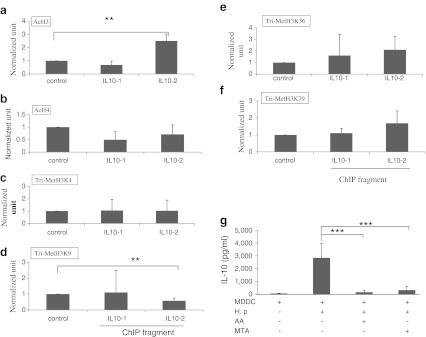
*H. pylori* regulates IL-10 production via histone modifications in healthy controls. A ChIP assay was used to analyze the relative levels of **a** acetylated H3 and **b** acetylated H4, **c** tri-methylated H3K4, **d** H3K9, **e** H3K36, and **f** H3K79 at the IL-10 gene promoter region of nine healthy individuals. **g** IL-10 production was significantly lower in MDDCs treated with the histone acetyltransferase inhibitor anacardic acid (*AA*) and the histone methyltransferase inhibitor MTA. *p <0.05, **p <0.01, ***p <0.001

Histone modification has been implicated as an important process for controlling gene expression (Li et al. 2007). To determine the effect of histone modification on IL-10 gene transcription, ChIP assays were performed. Histone H3 and H4 acetylation as well as H3K4, H3K9, H3K36, and H3K79 trimethylation were tested for regulating effects on IL-10 gene expression. The IL-10 promoter locus of *H. pylori*-treated MDDCs exhibited increased acetylation of H3 (Fig. 6a) and decreased trimethlyation of H3K9 (Fig. 6d). Histone acetylation has been reported to be associated with increase of gene transcription, while H3K9 trimethylation is related to the increase or decrease of gene transcription (Li et al. 2007). Our observations suggest that histone modification after stimulation with *H. pylori* is a prerequisite for IL-10 gene transcription. The involvement of histone acetylation and methylation in IL-10 expression was further supported by observations of MDDCs stimulated with *H. pylori* after 1-h treatment with histone acetyltransferases inhibitor AA and histone methyltransferase inhibitor MTA. Forty-eight hours after stimulation, the cell supernatants were harvested and tested for IL-10 production by ELISA. IL-10 production was significantly decreased in MDDCs treated with AA and MTA (Fig. 6g). These observations confirm the important role of *H. pylori* in controlling histone epigenetic modification to regulate IL-10 expression.

## Discussion

Recent studies indicate that the host immune response is related to the pathogenesis of gastric cancer (Bornschein et al. 2010; Suerbaum and Michetti 2002). Previous studies of the host response to *H. pylori* have focused on the role of macrophages (Keep et al. 2010), but the presence of DCs in the surface epithelium of the gastric mucosa and their recruitment during *H. pylori* infection suggest that DCs are critical in the host response to *H. pylori* infection.

Fully mature DCs upregulate co-stimulatory and adhesion molecules (e.g., CD40, CD54, CD80, and CD86) and MHC class II antigens, but decrease antigen uptake and processing (Langenkamp et al. 2000; Moser and Murphy 2000), which plays a key role in regulating innate and adaptive immunity. In contrast, immature DCs do not form stable DC–effector T cell contacts and prevent the effective priming of an immune response. Therefore, the presence of immature DCs may facilitate the persistence of pathogens and their ability to evade or subvert the host immune response (Ménétrier-Caux et al. 2009; Mahnke et al. 2007). In our present study, the observations of poor generation of the maturation marker CD40 on DCs following *H. pylori* infection and less IL-17 expression in gastric cancer patients indicated that immature DCs impaired the DCs’ capacity to induce autologous T-cell activation. Furthermore, the inhibitory potential of immature DCs in gastric cancer patients may be an explanation for the immune escape mechanism of this persistent pathogen during cancer development.

Regulatory T cells (Treg) and dendritic cells (DC) play an important role in tumor immunity and immune escape (Kandulski et al. 2010). Regulatory immune cells, mostly regulatory Foxp3^+^CD4^+^CD25^+^ T cells (Treg cells), have been identified as a major regulatory component of the adaptive immune response and are involved in *H. pylori*-related inflammation and bacterial persistence (Shen et al. 2010). Only mature/activated DCs stimulate T-cell proliferation. Immature DCs have tolerogenic properties and can induce nonproliferative Tregs and suppress proliferation of CD4+ and CD8+ effector T cells. There is growing evidence that expression of the Treg marker Foxp3 mRNA is higher in the gastric tissue of *H pylori*-infected individuals, a characteristic that may contribute to immune tolerance (Shen et al. 2010). Extensive Foxp3+ Treg infiltration and a high Foxp3+/CD8+ ratio are also associated with poor prognosis in gastric cancer (Shen et al. 2010). Schuler et al. reported that MDDC in advanced head and neck squamous cell carcinoma (HNSCC) patients show a maturation deficiency *ex vivo*, and these patients have a higher frequency of Tregs compared with healthy controls (*p* = 0.001) (Schuler et al. 2011).

Previous reports have stated that T regulatory cells (Tregs) suppress the inflammatory reaction driven by IL-17, thereby favoring bacterial persistence (Schuler et al. 2011; Velin et al. 2009). The pro-inflammatory cytokine IL-17 can induce severe inflammation with infiltration of neutrophils and is a key mediator in the host defense against bacterial organisms (Kabir 2011; Schuler et al. 2011). In an animal model, treatment with anti-IL-17 antibodies led to a significantly greater inhibition of inflammation and reduction of *Helicobacter* infection in comparison with control antibodies (Velin et al. 2009).

Our data show increased IL-17 expression in response to *H. pylori* in both healthy controls and gastric cancer patients, but significantly lower IL-17 in gastric cancer patients. We speculate that *H. pylori* infection induces immature MDDCs in gastric cancer patients, which may increase gastric regulatory T cell (Treg) responses. Furthermore, lower IL-17 expression in gastric cancer patients may be suppressed by Treg cells; it may drive Th17/Treg balance toward a Treg bias and lead to an ineffective host immune response to eradicate *H. pylori*. Prospective studies of Tregs in gastric cancer patients infected with *H. pylori* are necessary to assess these hypotheses.

The risk for developing cancer rises substantially as a result of poorly regulated inflammatory responses to pathogenic bacterial infections. Individuals with a weakened IL-10 mediated inhibitory loop are highly susceptible to the carcinogenic consequences of elevated inflammation and show more frequent inflammation-associated cancers. Previous studies have indicated that induction of an *H. pylori*-specific Th1 response is a crucial component of protective immunity against *H. pylori* (Eaton et al. 2001; Shi et al. 2010; Takeshima et al. 2009; Zhang et al. 2008). Individuals carrying genotypes associated with increased pro-inflammatory cytokine production and/or decreased anti-inflammatory cytokine IL-10 production reportedly have increased risk of gastric cancer. The Th2 cytokine IL-10 can down-regulate the host immune response by modulating the *Helicobacter*-specific Th1 immune response and reducing the severity of gastritis induced by *H. pylori* (Eaton et al. 2001).

In our present study, stimulation with *H. pylori* led to increased IL-10 production by MDDCs. However, IL-10 expression was significantly lower in MDDCs from gastric cancer patients as compared with healthy controls. We speculate that immature DCs in gastric cancer patients do not produce sufficient IL-10. Furthermore, the low IL-10 levels were insufficient to modulate the *Helicobacter*-specific Th1 immune response and may cause severe inflammation and hyperresponsiveness in the gastric lumen, leading to gastric diseases.

The mechanism of *H. pylori* recognition by DCs and the intracellular signaling pathways that regulate IL-10 expression are poorly understood. Rad et al. (2007, 2009) have identified a MyD88-dependent component of the DC activation program that is induced by surface TLRs recognizing *H pylori.* Mean et al. showed that *H. pylori* induces DC maturation through TLR binding (Means et al. 2003).

The C-type lectin DC-SIGN, which is a dendritic cell pathogen-associated molecular pattern (PAMP) receptor, participates in the recognition and capture of numerous viral, bacterial, and fungal pathogens (Geijtenbeek et al. 2004; Naarding et al. 2006). There are some studies reported the DC-SIGN receptor on DCs and its effects on cytokine regulation after *H. pylori* infection (Bergman et al. 2004; Gringhuis et al. 2009). Our results provide insight into the mechanisms of *H. pylori* recognition by DCs and indicate that regulation of IL-10 expression is mediated by TLR2, TLR4, and DC-SIGN receptors on DCs. Due to anti-DC-SIGN, TLR2, or TLR4 antibodies treatment alone blocked IL-10 production almost completely, therefore it was hard to observe the further enhanced effect when any antibody combination treatment was used. Nevertheless, the process whereby *H. pylori* binds to DCs is highly complex, and further study is needed to determine whether candidate receptors function alone or in concert with other surface components during this process.

Whether the MAPK intracellular signaling pathway mediates IL-10 expression in DCs infected with *H. pylori* is still unclear. NF-κB, a key coordinator of innate immunity and inflammation (Karin 2006), regulates the expression of genes encoding inflammatory cytokines such as IL-10. NF-κB is a heterodimeric protein which consists of a p50 and a p65 subunit; phosphorylation of p65 enables translocation of the p50–p65 complex to the nucleus, where it interacts with the transcriptional coactivators p300 and CBP, which contain histone acetyltransferase domain. Immune subversion by histone modification correlates with changes in host gene expression (Li et al. 2007). Our results show that DCs treated with *H. pylori* exhibit preferential activation of the p-38 MAPK pathway as well as differential effects on IL-10 expression associated with enhanced histone modifications at the IL-10 gene locus. Adding acetylation and methylation inhibitors ablated the IL-10 expression induced by *H. pylori*. These observations demonstrate that *H. pylori* regulates DC function via epigenetic modulation of IL-10 cytokine expression. Due to the limited availability of MDDCs from gastric cancer patients, intracellular events in *H. pylori* infected dendritic cells (DCs) from healthy individuals were evaluated in this study. Whether lower IL-10 production in gastric cancer patients than healthy controls results from the presence of immature DC or impaired intracellular signaling pathways merits evaluation in further work.

In conclusion, our study shows that MDDCs from gastric cancer patients produce significantly lower levels of IL-10, and show poor maturation and failure to induce a robust Th17 response in response to *H. pylori.* These data suggest that defective DC responses to *H. pylori* in gastric cancer patients may impair the elimination of *H. pylori* in early stages and precipitate the persistent colonization afterwards. The functional alterations of MDDCs may contribute to chronic infection and cancer development. In the presence of *H. pylori*, TLR/DC-SIGN positively regulates MDDC maturation and IL-10 cytokine production via the p38-MAPK signaling and histone modification.
